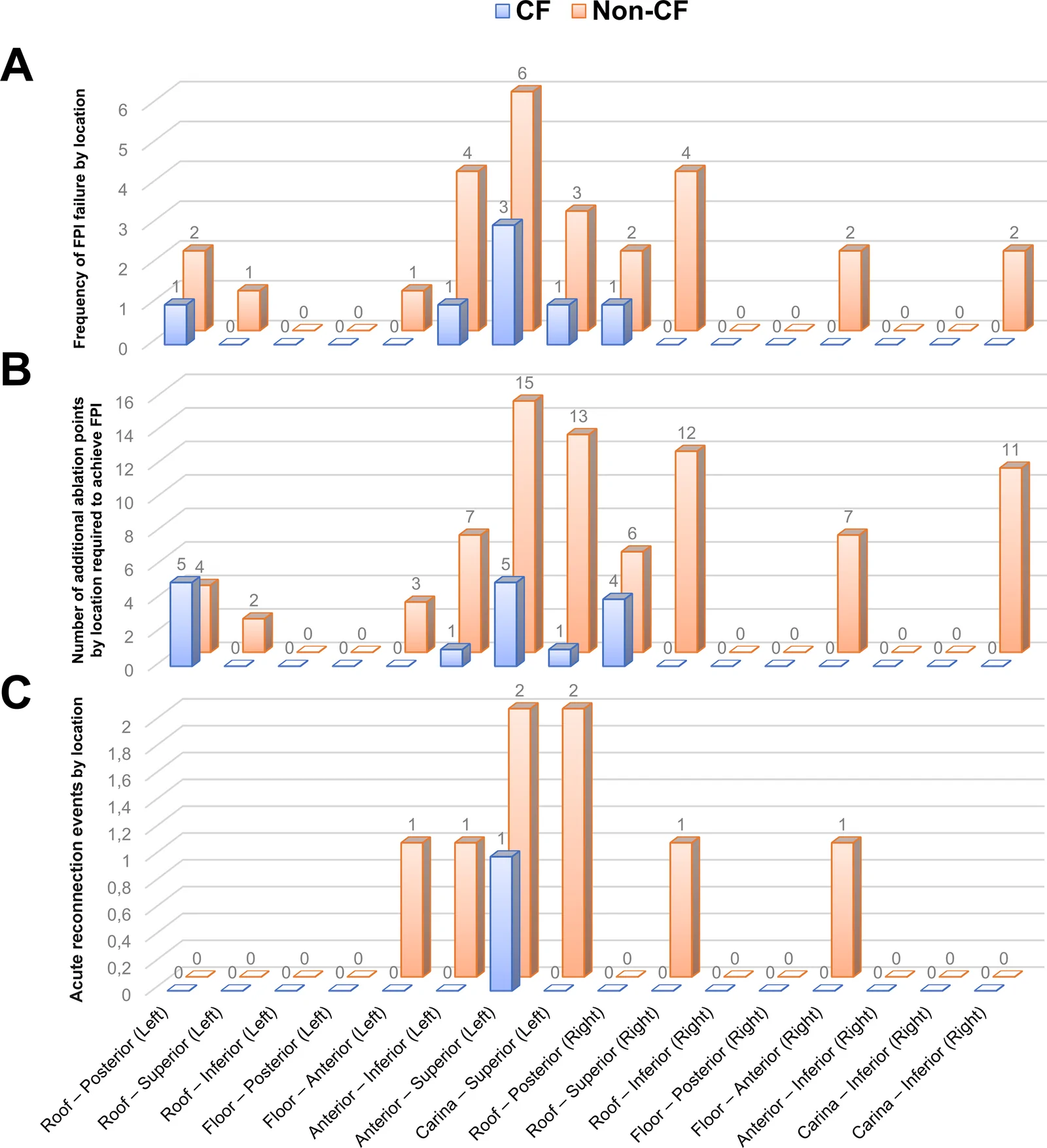# Evaluation of technical and economic outcomes of contact force-sensing catheters for pulmonary vein isolation in symptomatic paroxysmal atrial fibrillation in a developing country

**DOI:** 10.1016/j.ipej.2026.06.002

**Published:** 2026-06-03

**Authors:** Giang Song Tran, Tuan Le Van, Long Vien Hoang, Linh Tran Pham, Hai Minh Dang, Duc Minh Dao, Nhi Trinh Van, Thai Quoc Nguyen, Hoai Thi Thu Nguyen, Hai Nguyen Ngoc Dang, Thang Viet Luong, Quan Huynh, Thomas H. Marwick

**Affiliations:** a Vietnam National Heart Institute, Bach Mai Hospital, Ha Noi, Viet Nam; b Menzies Institute for Medical Research, University of Tasmania, Hobart, Australia; c Baker Heart and Diabetes Institute, Melbourne, Victoria, Australia

**Keywords:** Atrial fibrillation, Pulmonary vein isolation, Contact-force catheter, Catheter ablation, First-pass isolation, Developing country

## Abstract

**Introduction:**

Contact force (CF)-sensing catheters may improve lesion quality and procedural efficiency during pulmonary vein isolation (PVI) for paroxysmal atrial fibrillation (AF), but data on their clinical and economic implications in developing countries remain limited. This pilot study evaluated the technical, clinical, and economic outcomes of CF catheters compared with non-CF catheters in symptomatic paroxysmal AF.

**Methods:**

In this pilot study conducted in Vietnam, 45 patients underwent PVI. Technical outcomes included first-pass isolation (FPI), fluoroscopy time, and lesion-related procedural measures. Clinical outcomes included acute pulmonary vein reconnection and AF recurrence at 3 months. An economic analysis was performed from the payer perspective.

**Results:**

CF-guided PVI was associated with lower fluoroscopy time than non-CF ablation (12.19 ± 2.46 vs 14.04 ± 2.93 min, p = 0.026) and higher bilateral FPI (81.0% vs 37.5%, p = 0.003). The acute reconnection rate was 2.38% with CF and 8.33% with non-CF (p = 0.170). No procedural complications occurred in either group. At 3 months, 85.7% of patients in the CF group and 58.3% in the non-CF group were free from AF recurrence (p = 0.055). Total procedural cost was higher with CF catheters (201.08 ± 23.56 vs 191.03 ± 9.71 million VND), with an ICER of 36.7 million VND per additional patient free from AF recurrence at 3 months.

**Conclusion:**

CF-guided PVI was associated with better technical performance and a possible favorable short-term clinical trend, despite higher upfront costs. Exploratory economic analysis suggested a possible short-term economic benefit, pending confirmation in larger long-term studies.

## Highlights

• Contact force-sensing catheters for pulmonary vein isolation in symptomatic paroxysmal atrial fibrillation had better technical performance.
• This approach was associated with potentially more favorable short-term clinical outcomes.
• Contact force-guided pulmonary vein isolation suggested a possible short-term economic benefit.

## 1 Introduction

Atrial fibrillation (AF) is a common supraventricular arrhythmia with a growing global burden [1]. Beyond its high prevalence, AF is associated with major adverse outcomes such as heart failure and myocardial infarction. It also increases mortality and imposes a substantial medical and economic burden [2,3]. Accordingly, current guidelines recommend comprehensive management based on stroke prevention, rate or rhythm control, catheter ablation, and risk factor modification. Among symptomatic patients with paroxysmal AF, catheter ablation remains the most effective strategy for maintaining sinus rhythm [[3], [4], [5]]. However, in low- and middle-income countries such as Vietnam, access to catheter ablation remains limited because of restricted electrophysiology capacity, a shortage of trained operators, and financial barriers [6,7].

In Vietnam, when catheter ablation is performed, pulmonary vein isolation (PVI) through wide-area circumferential ablation remains the foundational strategy. However, most centers still rely on standard open-irrigated catheters. With earlier catheter technologies, achieving durable PVI was often time consuming and highly operator dependent, contributing to recurrent arrhythmia [8]. Because lesion durability depends on several procedural factors, optimizing lesion quality has become a key procedural goal. These factors include radiofrequency power, interlesion spacing, catheter stability, ablation duration, and tissue contact force [9]. Although three-dimensional electroanatomical mapping improves lesion spacing, tissue contact remains operator dependent. In this context, contact force (CF)-sensing catheters provide real-time, objective feedback on catheter-tissue contact and have been associated with better lesion quality, shorter procedures, and less fluoroscopy exposure [[10], [11], [12]].

Although CF technology has recently been introduced in Vietnam, its clinical and economic value in local practice remains uncertain. This is particularly relevant because higher catheter costs may affect hospital purchasing decisions in resource-constrained settings. We therefore sought to explore whether the technical and clinical advantages of CF-guided ablation might offset its additional short-term procedural cost. This pilot study compared the technical efficiency, clinical outcomes, and exploratory short-term economic findings of CF and non-CF catheters during PVI for symptomatic paroxysmal AF.

## 2 Methods

### 2.1 Study design

This pilot study was a prospective controlled interventional study with two parallel nonrandomized groups. It compared the technical performance and economic outcomes of CF-sensing and standard non-CF catheters during PVI for symptomatic paroxysmal AF. The study was conducted at the Vietnam National Heart Institute, Bach Mai Hospital, from July 2024 to July 2025. The protocol was approved by the Institutional Review Board of Ha Noi Medical University (approval number CKII37/GCN-HMUIRB) and was conducted in accordance with the 2013 Declaration of Helsinki. The study was reported in line with the TREND statement, and all patients provided written informed consent. As this was a pilot study, no formal sample size calculation was performed [13,14].

### 2.2 Participants

All consecutive patients aged eighteen years or older with symptomatic paroxysmal AF referred for catheter ablation were screened. Antiarrhythmic drugs were discontinued for at least five half-lives before the procedure, except for amiodarone. Eligible patients had paroxysmal AF without structural valvular disease and were considered appropriate candidates for catheter ablation according to the AF guidelines [4,5]. Patients had no contraindications to ablation and provided informed consent. The exclusion criteria included valvular heart disease, rheumatic valvular heart disease, acute deep vein thrombosis, intracardiac thrombus, active bleeding or coagulopathy, myocardial infarction within three months, and an international normalized ratio greater than three [4,5,15]**.**

### 2.3 Study procedures

All patients underwent structured clinical assessment, which included symptom evaluation, AF duration, European Heart Rhythm Association class, physical examination, and laboratory and imaging tests. Preprocedural investigations included an electrocardiogram, transthoracic echocardiography, transesophageal echocardiography to exclude left atrial (LA) thrombus, and computed tomography when indicated.

### 2.4 Electrophysiological study and catheter ablation

Procedures in both groups were performed by the same core electrophysiology operators. Standard multipolar catheter placement and transseptal access were used, followed by intravenous heparin. LA geometry was reconstructed with a three-dimensional electroanatomical mapping system (EnSite Precision™, Abbott) (Fig. 1), and wide-area circumferential ablation was performed around each pulmonary vein pair using either CF or non-CF catheters. Successful PVI was defined as complete bidirectional electrical isolation. If AF persisted after ablation, electrical cardioversion was performed, followed by reassessment of isolation.

**Fig. 1:**
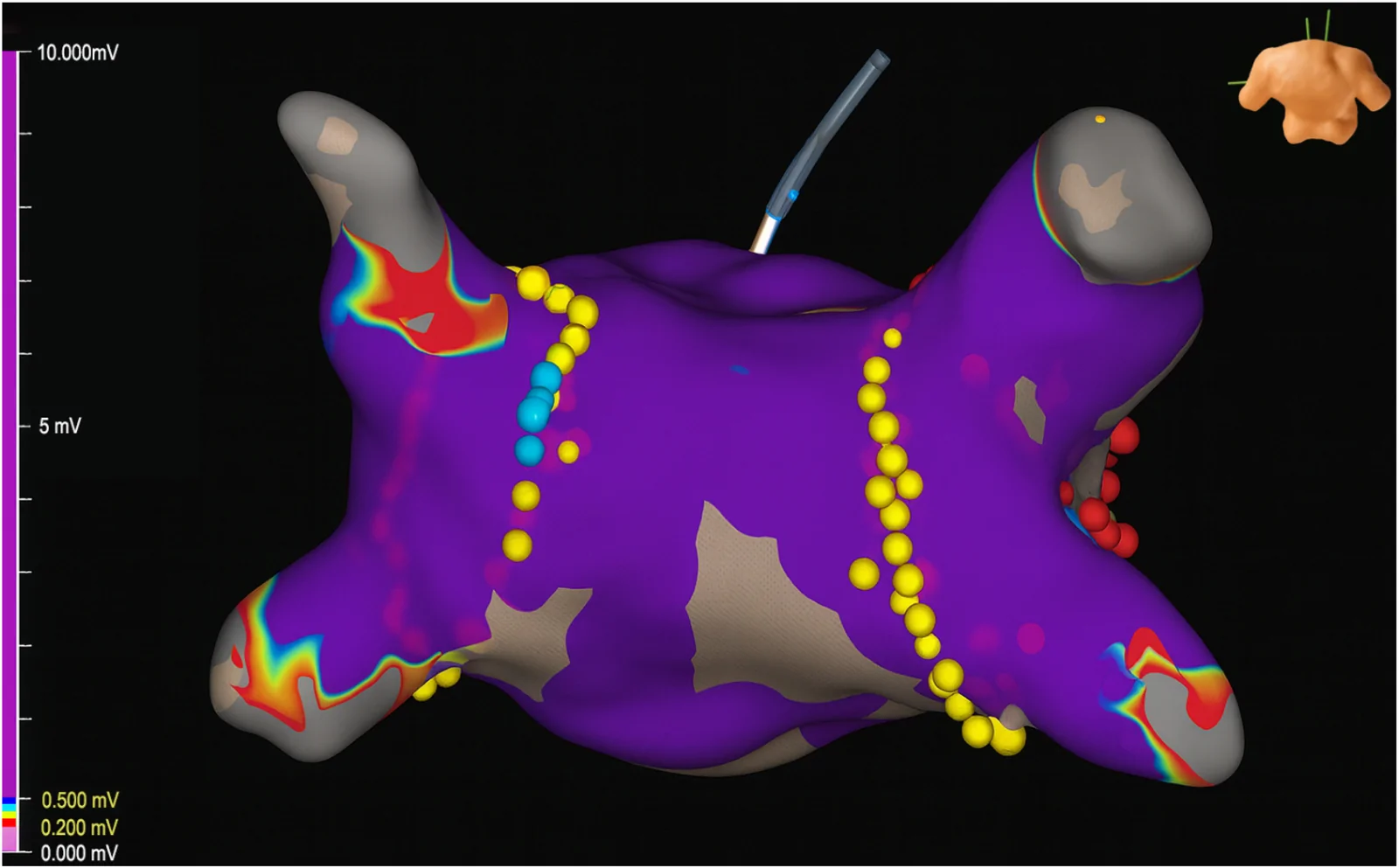
Three-dimensional electroanatomical mapping and left atrial ablation illustration.

Radiofrequency energy was delivered using a conventional point-by-point low-power strategy, with 35 W applied along the posterior wall and 40 W along the anterior wall. A high-power short-duration approach was not used. In the CF group, TactiCath™ catheters (Abbott) were used, and ablation was guided by lesion size index targets of 4.0 on the posterior wall and 5.0 on the anterior wall. In the non-CF group, lesion delivery was guided primarily by a fixed ablation duration of 30 s at each point after stable catheter positioning had been achieved. A decrease in impedance was used as supportive procedural feedback, and a reduction of approximately 10 Ω was considered a meaningful change when observed [16,17]. Intraprocedural and postprocedural complications were recorded.

### 2.5 Outcomes

The primary outcomes included successful bilateral first-pass pulmonary vein isolation (FPI) and AF recurrence during the 3-month follow-up. The secondary outcomes included procedural complications and total procedure duration. Patients were followed for three months via a 24-h Holter monitor. AF recurrence during follow-up was defined as any documented AF episode lasting at least 30 s [15]. FPI was defined as the achievement of complete bidirectional electrical isolation between the LA and the pulmonary vein (PV) immediately after completion of the circumferential ablation line, without the need for any additional ablation. In contrast, non-FPI refers to failure to achieve immediate bidirectional LA–PV block after the initial ablation circle, requiring additional radiofrequency applications to achieve PVI. A conduction gap was defined as a site of residual electrical connection between the LA and PVs identified after completion of the first-pass PVI. Acute procedural success was defined as a stable bidirectional electrical conduction block between the LA and PVs, confirmed 30 min after initial successful PVI. Acute reconnection after PVI was defined as the presence of LA–PV electrical reconduction detected during reassessment at the 30-min waiting period.

### 2.6 Economic analysis

The analysis was performed from the payer perspective, specifically the Vietnam Social Security system. Costs were limited to direct procedural hospitalization costs during the index ablation admission, and effectiveness was defined as freedom from AF recurrence at 3 months. A cost-effectiveness framework was chosen because the comparison involved both incremental costs and an incremental clinical outcome measured in natural units. Economic value was summarized using the incremental cost-effectiveness ratio (ICER), which represents the additional cost required to achieve one additional patient free from AF recurrence at 3 months with CF-guided ablation compared with the non-CF strategy [18,19]. A willingness-to-pay benchmark of one gross domestic product per capita was explored as a contextual scenario [20,21], equivalent to 4717 USD or 117.9 million VND at the time of the study [22]. In this framework, CF-guided ablation was considered to show a potentially favorable economic signal when the ICER was lower than the contextual willingness-to-pay benchmark, although uncertainty around the estimate was also considered in interpretation.

### 2.7 Statistical analysis

All the statistical tests were two-sided, with the significance set at p < 0.05. Analyses were performed via R Studio version 4.4.1. Patients with missing data were excluded. The distribution of continuous variables was assessed via the Shapiro–Wilk test. Normally distributed variables are expressed as the means and standard deviations and were compared via Student's *t*-test. Nonnormally distributed variables are presented as medians and interquartile ranges and were compared via the Wilcoxon–Mann–Whitney test. Categorical variables were compared using the chi-square test or Fisher's exact test, as appropriate. For the economic analysis, nonparametric bootstrapping with 5000 iterations was used to estimate uncertainty around incremental cost-effectiveness results and to generate cost-effectiveness planes. Cost-effectiveness acceptability curves (CEAC) were constructed to estimate the probability that CF would be cost-effective across a range of willingness-to-pay thresholds. In addition, one-way sensitivity analyses were performed.

## 3 Results

### 3.1 Baseline patient characteristics

In this pilot cohort of forty-five patients, no significant differences were observed between the two groups in terms of anthropometric variables, medical history, comorbidities, or clinical presentation. The detailed baseline characteristics are presented in Table 1 and Supplementary Table S1.

**Table 1:** Baseline characteristics of the study population.

Variable	CF (n = 21)	Non-CF (n = 24)	P value
Age (years)	59.71 ± 10.37	61.04 ± 9.74	0.673
Female (%)	7 (33.3%)	10 (41.7%)	0.565
BMI (kg/m^2^)	23.40 ± 1.79	23.19 ± 2.22	0.720
**Medical history & comorbidities**			
Heart failure (%)	6 (28.6%)	7 (29.2%)	0.965
Hypertension (%)	12 (57.1%)	16 (66.7%)	0.511
Type 2 diabetes (%)	1 (4.8%)	6 (25.0%)	0.101
Smoking (%)	11 (52.4%)	11 (45.8%)	0.661
**Clinical status**			
EHRA class	2.00 [2.00–3.00]	2.00 [2.00–2.25]	0.731
Heart rate (beats/min)	80.00 [70.00–90.00]	79.00 [70.00–90.00]	0.673
**Echocardiography**			
LVEF (%)	65.00 [61.00–67.00]	65.00 [61.75–68.00]	0.801
LA diameter (mm)	34.10 ± 3.69	36.00 ± 4.73	0.137
LAVI (mL/m^2^)	28.67 ± 6.12	31.67 ± 7.93	0.160
**Electrocardiography**			
P-wave duration (ms)	96.00 [89.00–110.00]	90.00 [90.00–100.00]	0.887
PR interval (ms)	156.67 ± 17.02	161.74 ± 14.25	0.283
QRS duration (ms)	90.00 [88.00–94.00]	90.00 [81.00–97.50]	>0.99
QT interval (ms)	390.00 [388.00–410.00]	400.00 [389.00–426.50]	0.135

The data are presented as means ± standard deviations, medians [interquartile ranges], or numbers (percentages), as appropriate. Abbreviations: BMI, body mass index; CF, contact force (catheter); Non-CF, non-contact force (catheter); EHRA, European Heart Rhythm Association; LVEF, left ventricular ejection fraction; LA, left atrium; LAVI, left atrial volume index.

### 3.2 Technical and clinical outcomes

Table 2 summarizes the technical outcomes in the two groups. Preparation-to-transseptal time was shorter in the CF group (35 min vs 40 min, p = 0.017), whereas 3D mapping time was longer (20 min vs 15.5 min, p = 0.047). Total fluoroscopy time was lower in the CF group (12.19 ± 2.46 min vs 14.04 ± 2.93 min, p = 0.026). The total number of ablation lesions did not differ significantly between the groups. FPI rates were higher in the CF group for both the left veins (85.7% vs 50.0%, p = 0.011) and the right veins (95.2% vs 62.5%, p = 0.012). Total procedure time was shorter in the CF group than in the non-CF group (160.00 [150.00–170.00] vs 170.00 [160.00–192.50], p = 0.015). Bilateral FPI was also more frequent in the CF group (81.0% vs 37.5%). The acute electrical reconnection rate did not differ significantly between the groups (2.38% in the CF group vs 8.33% in the non-CF group, p = 0.170), as shown in Supplementary Figure S1.

**Table 2:** Comparison of technical outcomes between the CF and non-CF groups.

Variable	CF (n = 21)	Non-CF (n = 24)	P value
**Total procedure time (minutes)**	160.00 [150.00–170.00]	170.00 [160.00–192.50]	0.015
**Preparation-to-transseptal time (minutes)**	35.00 [30.00–35.00]	40.00 [30.00–50.00]	0.017
**Posttransseptal fluoroscopy time (minutes)**	7.00 [6.00–8.00]	7.00 [5.75–8.00]	0.982
**Total fluoroscopy time (minutes)**	12.19 ± 2.46	14.04 ± 2.93	0.026
**3D mapping time (minutes)**	20.00 [16.00–20.00]	15.50 [15.00–20.00]	0.047
**Left ablation lesions (n)**	32.00 [24.00–34.00]	32.50 [26.75–46.75]	0.106
**Right ablation lesions (n)**	35.00 [30.00–38.00]	34.00 [29.75–49.75]	0.387
**Total ablation lesions (n)**	63.00 [56.00–73.00]	71.50 [60.50–96.25]	0.088
**Left first-pass isolation (%)**	18 (85.7%)	12 (50.0%)	0.011
**Right first-pass isolation (%)**	20 (95.2%)	15 (62.5%)	0.012
**Bilateral first-pass isolation (%)**	17 (81.0%)	9 (37.5%)	0.003
**Tissue impedance (Ω)**	114.48 ± 8.34	106.17 ± 8.45	0.004
**Blood impedance (Ω)**	99.00 ± 9.31	92.33 ± 8.50	0.031
**Complication (%)**	0 (0)	0 (0)	>0.99

The data are presented as the means ± standard deviations, medians [interquartile ranges], or numbers (percentages), as appropriate. Abbreviations: CF, contact force (catheter); Non-CF, non-contact force (catheter).

Supplementary Fig. S2 shows that failure to achieve FPI occurred predominantly in the non–contact force (non-CF) group, particularly in the left anterior–superior and left anterior–inferior regions. In contrast, the CF group exhibited no more than three failures at the same anatomical locations. The number of additional lesions required to achieve FPI was also substantially greater in the non-CF group, reaching fifteen lesions in the left anterior–superior region and thirteen lesions in the left carina–superior region, whereas the maximum number in the CF group was five lesions. Acute site-specific reconnection was observed mainly in the non-CF group, most frequently in the left anterior–superior region and left carina–superior, whereas the CF group demonstrated very few events with a scattered distribution.

Regarding clinical follow-up, freedom from AF recurrence at 3 months was higher in the CF group than in the non-CF group, with rates of 85.7% and 58.3%, respectively. The corresponding numbers were 18 of 21 patients and 14 of 24 patients, as shown in Supplementary Table S2. However, this difference did not reach statistical significance (p = 0.055).

### 3.3 Economic outcomes

**Fig. 2:**
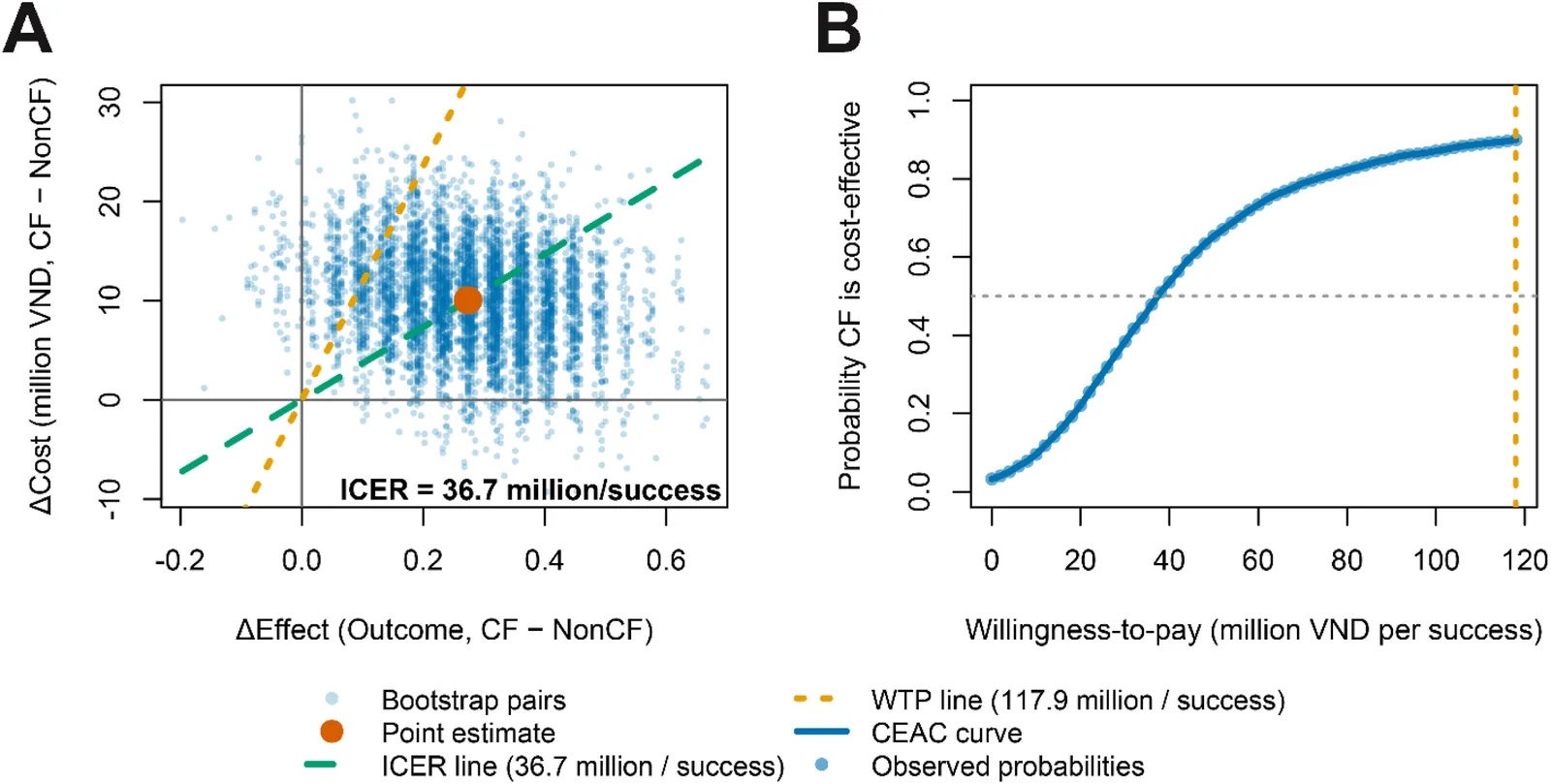
**Exploratory economic analysis comparing the CF and non-CF groups.** Panel A shows the cost-effectiveness plane for freedom from AF recurrence at 3 months. Panel B shows the corresponding cost-effectiveness acceptability curve. Abbreviations: CEAC, cost-effectiveness acceptability curve; CF, contact force; ICER, incremental cost-effectiveness ratio; non-CF, non-contact force; VND, Vietnamese dong; WTP, willingness-to-pay; Δ, difference.

The economic analysis used freedom from AF recurrence at 3 months as the effectiveness outcome. The mean total procedural cost was higher in the CF group than in the non-CF group (201.08 ± 23.56 million VND vs 191.03 ± 9.71 million VND), corresponding to an incremental cost of 10.05 million VND per patient. Based on these differences, the incremental cost-effectiveness ratio was 36.7 million VND per additional patient free from AF recurrence at 3 months. The ICER point estimate was below the contextual willingness-to-pay benchmark of 117.9 million VND. However, the bootstrap 95% confidence interval was wide, ranging from −7.19 to 227.32 million VND. Fig. 2 illustrates the uncertainty around this estimate through the cost-effectiveness plane and acceptability curve, while Supplementary Table S2 provides the corresponding bootstrap confidence interval and one-way sensitivity analyses.

## 4 Discussion

This pilot study provides initial evidence on the technical performance, clinical outcomes, and exploratory short-term economic findings of CF-guided PVI for symptomatic paroxysmal AF in Vietnam. In this prospective controlled nonrandomized study of 45 patients, early technical outcomes favored the CF group. CF technology appeared to improve lesion quality and acute procedural performance. The CF group achieved a significantly higher FPI rate, which likely reflects more stable catheter contact and the creation of deeper, more continuous lesions with fewer conduction gaps. In contrast, lesion delivery in the non-CF group depended more heavily on operator perception, which may increase the risk of shallow or nontransmural lesions. Anatomically, contact force tended to be lower at the left-sided segments, a region that appears particularly vulnerable to incomplete lesion formation and reconnection. This pattern is supported by the clustering of recurrent activity in the left anterior-superior region and is consistent with prior international reports [23,24]. Acute reconnection did not differ significantly between groups, although the observed rate was lower in the CF group. The recurrence-free rate at 3 months was also higher in the CF group, suggesting a possible favorable clinical trend. However, given the pilot design, limited sample size, and short follow-up, these findings are not sufficient to confirm a clinical benefit.

In our study, total procedure time was shorter in the CF group than in the non-CF group. However, this finding likely reflects only part of the technical advantage associated with CF guidance. Part of the difference was related to a longer preparation-to-transseptal phase in the non-CF group. In our study, several non-CF cases were associated with technically more difficult coronary sinus catheter placement and other early preparatory steps. These events appeared to be incidental rather than systematic, but they happened to occur in the non-CF group and contributed to prolongation of the initial procedural phase. By contrast, 3D mapping time was longer in the CF group. In our setting, all procedures were performed under local anesthesia, and discomfort during ablation sometimes led to deep breathing or body movement. This could shift the left atrial geometry and necessitate repeated remapping [25,26]. In the CF group, real-time contact-force feedback made loss of effective tissue contact easier to recognize. As a result, geometric mismatch may have been detected more readily, prompting supplementary remapping rather than continuation with a potentially inaccurate map. In the non-CF group, such map displacement may have been less apparent during the procedure. Furthermore, posttransseptal fluoroscopy time was similar between groups. This may be explained by the fact that these were the initial cases performed with CF technology, during which the operators still tended to maintain their previous fluoroscopic habits during left atrial manipulation in order to maximize procedural safety. Nevertheless, total fluoroscopy time remained lower in the CF group. Taken together with the higher FPI rate and the lower need for additional lesions, these findings suggest that CF guidance may have improved lesion-related procedural efficiency once adequate geometry had been established, although the overall time metrics were also influenced by preparation-related factors, remapping, and early operator learning. These findings are consistent with previous studies showing shorter procedure times and lower fluoroscopy exposure with CF-guided ablation than with non-CF approaches [11,27]. In many centers, near-zero fluoroscopy has now been achieved with modern electroanatomical mapping workflows, often with the support of intracardiac echocardiography [28].

Economic considerations are especially important in resource-limited settings. Previous studies have suggested that catheter ablation may remain economically attractive because of reductions in long-term healthcare utilization, and larger international trials have also supported the economic value of ablation and CF-guided strategies [[29], [30], [31]]. In our study, the total procedural cost was higher in the CF group. However, from the payer perspective, CF was associated with a higher rate of freedom from AF recurrence at 3 months, resulting in a lower incremental cost per additional recurrence-free patient than the contextual benchmark used in this study. This finding may be relevant in Vietnam, where adoption of new electrophysiological technologies is influenced by reimbursement limitations, hospital budgets, and the availability of local comparative data. It therefore provides useful local evidence for institutional decision-making.

### 4.1 Limitations

This study has several limitations. First, it was conducted at a single center in Vietnam, which limits its external generalizability. Second, the follow-up duration was short, and AF recurrence was assessed using a single 24-h Holter recording, so intermittent, late-onset, or long-term recurrence patterns may have been missed. Third, sedation practices vary across countries [32]. In our study, all patients remained fully conscious and received only local anesthesia. Pain, vagal reactions, respiratory motion, repeated remapping, and difficulty in positioning the coronary sinus catheter may have prolonged procedure time in both groups. These conditions may not reflect all clinical settings. Moreover, the experience of the core electrophysiology operators may have influenced the results, thereby limiting representativeness. Fourth, the economic analysis was exploratory and limited to direct procedural hospitalization costs during the index admission, using freedom from AF recurrence at 3 months as a short-term natural clinical outcome rather than a utility-based measure. Therefore, the willingness-to-pay threshold should be interpreted as a contextual benchmark rather than a definitive decision threshold. Indirect elements, such as opportunity costs and broader societal impact, were not evaluated. Fifth, catheter availability was influenced by procurement and system-related factors, so the study was necessarily nonrandomized and may have been confounded by temporal trends and operator learning effects. Future studies should incorporate multicenter data, longer follow-up, more comprehensive rhythm monitoring, and both direct and indirect economic components.

## 5 Conclusions

CF-guided PVI was associated with better technical performance and greater procedural efficiency. Although the upfront procedural cost was higher, the exploratory economic analysis suggested a possible short-term economic benefit. These findings support further evaluation of CF technology for AF ablation in Vietnam, a developing country where this approach is being progressively implemented. Larger studies are needed to confirm its long-term clinical and economic benefits.

## Consent to participate

Written informed consent to participate was obtained from all individual participants included in the study.

## Consent for publication

Not applicable.

## Availability of data and materials

The datasets used and/or analyzed during the current study are available from the corresponding author upon reasonable request.

## Ethics approval

The study protocol was approved by the Institutional Review Board of Ha Noi Medical University (approval number: CKII37/GCN-HMUIRB) and was conducted in accordance with the Declaration of Helsinki (2013 version).

## Author contributions

Conceptualization: Tuan Le Van, Giang Song Tran, Hai Nguyen Ngoc Dang, Thang Viet Luong;

Methodology: Tuan Le Van, Giang Song Tran, Hai Nguyen Ngoc Dang;

Formal analysis: Tuan Le Van, Giang Song Tran, Hai Nguyen Ngoc Dang;

Data curation: Tuan Le Van, Giang Song Tran, Long Vien Hoang, Linh Tran Pham, Hai Minh Dang, Duc Minh Dao, Nhi Trinh Van, Thai Quoc Nguyen, Hoai Thi Thu Nguyen;

Investigation: Tuan Le Van, Giang Song Tran, Long Vien Hoang, Linh Tran Pham, Hai Minh Dang, Duc Minh Dao, Nhi Trinh Van, Thai Quoc Nguyen, Hoai Thi Thu Nguyen;

Writing – original draft: Tuan Le Van, Giang Song Tran, Hai Nguyen Ngoc Dang, Thang Viet Luong;

Writing – review & editing: Hai Nguyen Ngoc Dang, Thang Viet Luong, Quan Huynh, Thomas H. Marwick;

Supervision: Hai Nguyen Ngoc Dang, Thang Viet Luong, Quan Huynh, Thomas H. Marwick;

Resources: Hai Nguyen Ngoc Dang;

Visualization: Tuan Le Van, Giang Song Tran;

Funding acquisition: Not applicable.

## Funding

This research received no external funding.

## Declaration of competing interest

The authors declare that they have no known competing financial interests or personal relationships that could have appeared to influence the work reported in this paper.

## Figures and Tables

**figs1:**
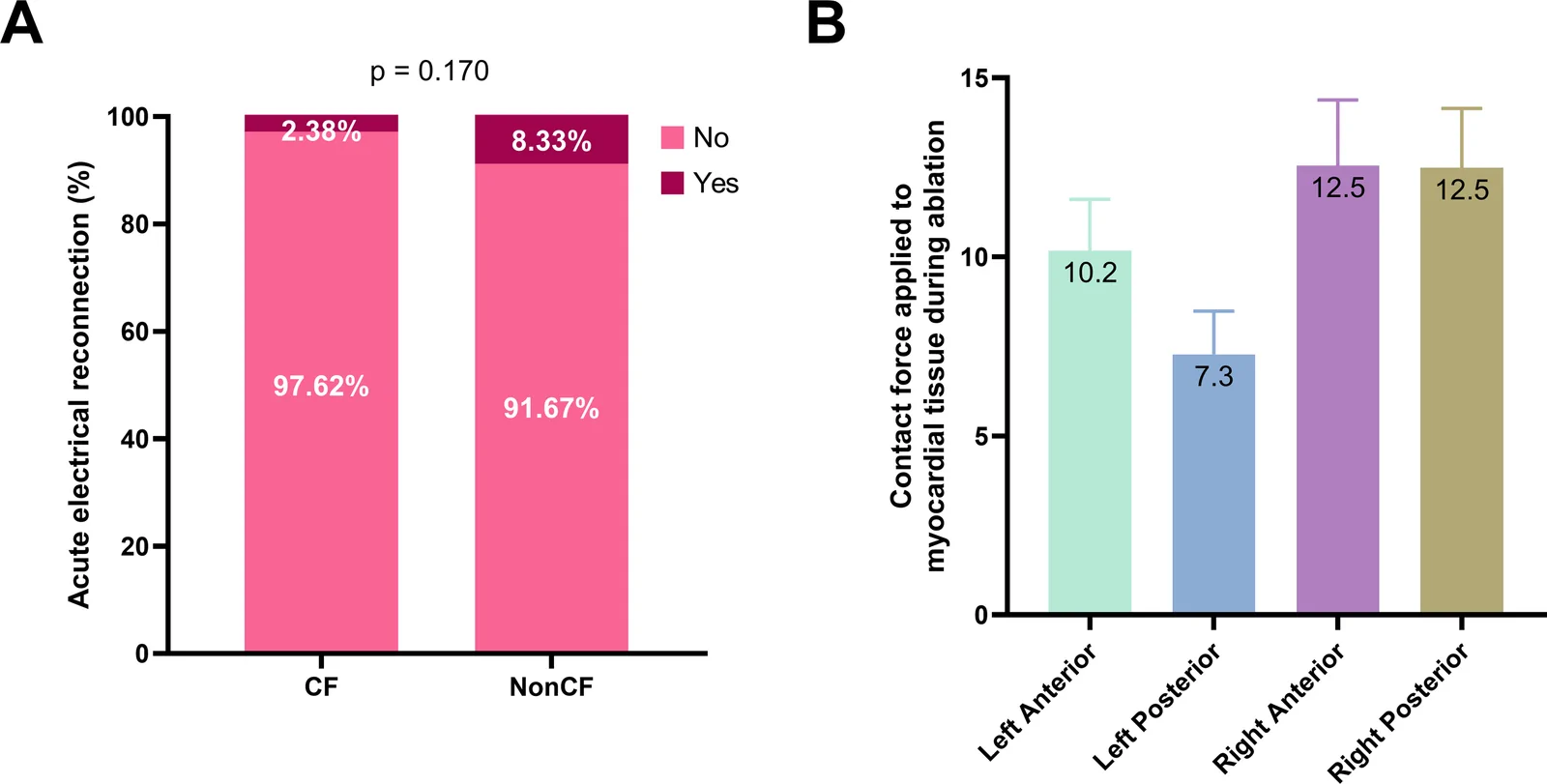


**figs2:**