# Supplementary material for: Evaluation of technical and economic outcomes of contact force-sensing catheters for pulmonary vein isolation in symptomatic paroxysmal atrial fibrillation in a developing country
Source: Indian Pacing Electrophysiol J. 2026 Jun 3;26(4):351–6. doi: 10.1016/j.ipej.2026.06.002 (PMC13541571; doi:10.1016/j.ipej.2026.06.002)
Supplement: Supplementary file 1 [file mmc1.docx]

**Supplementary Table S1. Baseline clinical and laboratory characteristics of the study population.**

| **Variable** | **CF (n=21)** | **Non-CF (n=24)** | **P value** |
| --- | --- | --- | --- |
| **Clinical status** | | | |
| SBP (mmHg) | 130.00 [120.00–135.00] | 120.00 [110.00–130.00] | 0.060 |
| DBP (mmHg) | 80.00 [70.00–80.00] | 80.00 [70.00–80.00] | 0.518 |
| CHA₂DS₂-VASc score (points) | 1.00 [0.00–2.00] | 1.00 [1.00–2.00] | 0.482 |
| HAS-BLED score (points) | 1.00 [0.00–1.00] | 1.00 [0.00–1.00] | 0.337 |
| **Laboratory tests** | | | |
| Urea (mmol/L) | 5.50 [5.30–5.70] | 5.40 [4.43–6.22] | > 0.99 |
| Creatinine (µmol/L) | 79.00 [68.00–90.00] | 80.00 [66.50–86.00] | 0.776 |
| AST (U/L) | 24.19 ± 9.17 | 23.54 ± 7.07 | 0.794 |
| ALT (U/L) | 24.00 [19.00–29.00] | 24.50 [19.50–32.25] | 0.577 |
| Free T4 (pmol/L) | 17.47 ± 1.51 | 16.15 ± 2.64 | 0.173 |
| Fasting glucose (mmol/L) | 5.10 [4.79–6.35] | 5.40 [5.10–7.30] | 0.184 |
| Troponin-T (ng/L) | 5.39 [4.19–8.66] | 7.38 [6.93–8.62] | 0.052 |
| NT-proBNP (pg/mL) | 106.00 [74.30–315.00] | 204.00 [25.95–244.50] | 0.798 |
| Sodium (mmol/L) | 140.33 ± 2.03 | 140.62 ± 2.14 | 0.642 |
| Potassium (mmol/L) | 3.95 ± 0.34 | 3.80 ± 0.32 | 0.143 |
| Red blood cell count (T/L) | 4.99 [4.65–5.22] | 4.78 [4.36–5.24] | 0.306 |
| Hemoglobin (g/L) | 137.86 ± 16.69 | 140.83 ± 14.04 | 0.524 |
| White blood cell count (×10⁹/L) | 7.06 ± 2.04 | 8.09 ± 2.09 | 0.103 |
| Platelet count (×10⁹/L) | 237.00 ± 52.52 | 262.29 ± 74.46 | 0.191 |

The data are presented as the means ± standard deviations, medians [interquartile ranges], or numbers (percentages), as appropriate. Abbreviations: SBP, systolic blood pressure; DBP, diastolic blood pressure; AST, aspartate aminotransferase; ALT, alanine aminotransferase; NT-proBNP, N-terminal pro-B-type natriuretic peptide; CF, contact force (catheter); Non-CF, non-contact force (catheter).
