# Supplementary material for: Evaluation of technical and economic outcomes of contact force-sensing catheters for pulmonary vein isolation in symptomatic paroxysmal atrial fibrillation in a developing country
Source: Indian Pacing Electrophysiol J. 2026 Jun 3;26(4):351–6. doi: 10.1016/j.ipej.2026.06.002 (PMC13541571; doi:10.1016/j.ipej.2026.06.002)
Supplement: Supplementary file 2 [file mmc2.docx]

**Supplementary Table S2. Cost and effectiveness outcomes for 3-month freedom from atrial fibrillation recurrence and corresponding incremental cost-effectiveness analyses.**

| **Cost and effectiveness outcomes used in the incremental cost-effectiveness analysis (VND)** | | | | | |
| --- | --- | --- | --- | --- | --- |
|  | **CF (n=21)** | | **Non-CF (n=24)** | | **P value** |
| **Effects** | 18 (85.7) | | 14 (58.3) | | 0.055 |
| **Total cost** | 201,077,955 ± 23,563,616 | | 191,031,875 ± 9,712,455 | | 0.007 |
| **Incremental cost-effectiveness ratios and one-way sensitivity analyses (million VND)** | | | | | |
| **ICER point estimate** | **95% CI** | **Parameter varied** | **Range (low–high)** | **ICER at low** | **ICER at high** |
| 36.7 | (−7.19 – 227.32) | Cost non-CF | 187 – 195 | 50.7 | 23.1 |
|  |  | Effect non-CF | 0.375 – 0.792 | 20.8 | 153.0 |
|  |  | Cost CF | 191 – 211 | 0.62 | 72.4 |
|  |  | Effect CF | 0.714 – 1.000 | 76.7 | 24.1 |

Data are presented as mean ± standard deviation or n (%), as appropriate. The effect outcome was defined as freedom from atrial fibrillation recurrence at 3 months. All costs are presented in Vietnamese dong (VND). The ICER represents the additional cost required to achieve one additional patient free from atrial fibrillation recurrence at 3 months with CF-guided ablation compared with the non-CF strategy. Bootstrap analysis was based on 5,000 iterations. One-way sensitivity analyses were performed by varying each parameter across the specified low-to-high range while holding the remaining parameters constant. Abbreviations: CF, contact-force catheter; Non-CF, non-contact force catheter; ICER, incremental cost-effectiveness ratio; CI, confidence interval.
